# Extraction of DNA from plant and fungus tissues *in situ*

**DOI:** 10.1186/1756-0500-5-266

**Published:** 2012-06-06

**Authors:** Amal S Abu Almakarem, Katie L Heilman, Heather L Conger, Yury M Shtarkman, Scott O Rogers

**Affiliations:** 1 Department of Biological Sciences, Bowling Green State University, Bowling Green, OH 43403, USA

**Keywords:** DNA extraction, Battery-operated microcentrifuge, Manually-operated centrifuge

## Abstract

**Background:**

When samples are collected in the field and transported to the lab, degradation of the nucleic acids contained in the samples is frequently observed. Immediate extraction and precipitation of the nucleic acids reduces degradation to a minimum, thus preserving accurate sequence information. An extraction method to obtain high quality DNA in field studies is described.

**Findings:**

DNA extracted immediately after sampling was compared to DNA extracted after allowing the sampled tissues to air dry at 21°C for 48 or 72 hours. While DNA extracted from fresh tissues exhibited little degradation, DNA extracted from all tissues exposed to 21°C air for 48 or 72 hours exhibited varying degrees of degradation. Yield was higher for extractions from fresh tissues in most cases. Four microcentrifuges were compared for DNA yield: one standard electric laboratory microcentrifuge (max rcf = 16,000×g), two battery-operated microcentrifuges (max rcf = 5,000 and 3,000 ×g), and one manually-operated microcentrifuge (max rcf = 120×g). Yields for all centrifuges were similar. DNA extracted under simulated field conditions was similar in yield and quality to DNA extracted in the laboratory using the same equipment.

**Conclusions:**

This CTAB (cetyltrimethylammonium bromide) DNA extraction method employs battery-operated and manually-operated equipment to isolate high quality DNA in the field. The method was tested on plant and fungus tissues, and may be adapted for other types of organisms. The method produced high quality DNA in laboratory tests and under simulated field conditions. The field extraction method should prove useful for working in remote sites, where ice, dry ice, and liquid nitrogen are unavailable; where degradation is likely to occur due to the long distances between the sample site and the laboratory; and in instances where other DNA preservation and transportation methods have been unsuccessful. It may be possible to adapt this method for genomic, metagenomic, transcriptomic and metabolomic projects using samples collected *in situ*.

## Background

Almost all methods to extract nucleic acids must be performed in a laboratory e.g., [1-8]. Many systematic, phylogenetic, molecular and related studies of plants and fungi utilize DNA and/or RNA as a primary source of data [9-14]. In most instances, fresh tissues are used for extraction of the nucleic acids, because degradation and other biochemical processes begin immediately after the tissue has been removed from the organism or from its natural substrate. This limits studies to plants and fungi that are in proximity to a research laboratory, including those that can be grown in greenhouses and/or growth chambers. However, a large number of research studies are conducted on organisms that must be collected in the field.

Three methods have been developed to preserve DNA in plant samples collected in the field [15-18]. One employs silica gel as a desiccant to rapidly dry the tissue, which reduces degradation in most specimens [15,18]. However, it does not eliminate degradation, and DNA yields are low for some tissues [19]. The second method uses a saturated NaCl-CTAB (i.e., brine-cetyltrimethylammonium bromide) solution [18]. The high salt partially dehydrates the tissues and the CTAB can complex with nucleic acids, proteins and carbohydrates to slow the degradation processes. However, high degrees of degradation have been noted in some cases with this method, and occasionally low yields of DNA result [19]. Addition of ascorbate mitigates some of the degradation. The third method uses an absorbent paper for preserving the DNA [16]. Pieces of plant or fungal tissue are smashed onto the paper, and then allowed to dry. Later, disks of the paper can be used (and reused) for PCR (polymerase chain reaction) amplification. While this is probably appropriate for many purposes, degradation has been reported [16]. Therefore, other means of preservation and/or extraction may be needed. DNA extraction at the site of sampling (i.e. *in situ*) is a possible alternative that can minimize degradation and maximize yield.

Degradation can be monitored by gel electrophoresis because as the DNA is broken down the higher molecular weight bands become more diffuse and smaller fragments of DNA are seen as increasingly bright smears of fluorescence extending into lower molecular weight regions of the gel see e.g. [8]. However, this only indicates that scission of the DNA strands is occurring. Simultaneously, other degradative processes also are occurring, resulting in losses of sequence information [20,21]. The most common changes are losses of bases by hydrolytic attack of the glycosidic bonds. Depurination occurs most often, but depyrimidization also occurs at a lower rate. When these DNAs are used as templates for PCR, approximately 75% of the time, an inaccurate base will be incorporated at those sites, causing a potential loss in sequence accuracy. Deamination of m^5^C (5-methylcytosine, prevalent in rRNA gene loci) produces a thymidine, which will pair with an adenosine rather than a guanosine during PCR amplification and sequencing reactions. Deamination of adenosine leads to the formation of hypoxanthine that will pair with a cytosine rather than a thymidine, again potentially causing loss of sequence accuracy. In our previous studies, we demonstrated that damaged DNA can be successfully amplified and sequenced, but often errors in the sequences are evident [22-24].

Significant DNA degradation can be observed shortly after removal of leaves or fungal tissue from the body of the primary organism or substrate [8], and RNA degradation is apparent within seconds after sampling. Some RNAs have half-lives of minutes to hours, while DNA degradation is a slower process. DNA in many species of plants and fungi has been detected in dried tissues from months to centuries after the organism has died [20,25]. Although DNA can be detected in tissues that have been dried for long periods of time (up to hundreds of years for fungi and up to tens of millennia for plants), degradation can be observed relatively soon after the cells begin to die [3,8,10-12,26]. Often, PCR amplification and sequencing can be accomplished from DNA extracted from tissues that have been dead for centuries to millennia. While DNA is present, can be detected, extracted, amplified and sequenced long after the plant or fungus tissue has been removed from the plant or basidiocarp, it is partially fragmented and otherwise altered by degradative enzymes, hydrolysis, oxidation and other processes, often leading to altered sequence results [20,21]. These processes increase in hot humid climates and vary with species. Therefore, sampling in such conditions at remote field sites may limit the utility of the extracted nucleic acids for molecular studies, unless extraction proceeds soon after sampling. Speed is of paramount importance to assure extraction of high quality DNA that is necessary to assure accurate and reproducible results. When the samples cannot be effectively sampled, preserved and transported rapidly to the laboratory, then alternatively the laboratory equipment and solutions can be transported to the target specimens in their natural environments in order to extract the DNA *in situ*.

**Figure 1:**
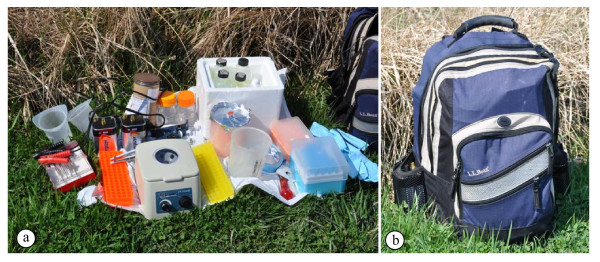
**Basic field DNA extraction equipment (a) and backpack (b).** All of the equipment needed for extraction to the point of DNA precipitation in ethanol is included. **a.** Equipment pictured (left to right): plastic waste containers, dissecting tools, adapter cables, 6 V batteries, microfuge tube racks, mailing canister (chloroform bottle inside), pipetters (P20, P200, P1000), battery-operated microfuge, 100% ethanol, 80% ethanol, styrofoam box (with 2X CTAB, CTAB precipitation buffer, high salt TE buffer, 0.1X TE, RO water), 1.5 ml microfuge tubes, waste container, labeling tape, manual grinding tools, pipette tips, gloves, backpack. Not pictured: heating source, manually-operated centrifuge (usually, either the battery-operated or the manually-operated centrifuge would be utilized; see Figure 2), and battery-operated grinding tools (see Figure 6). **b.** Backpack with all of the equipment and supplies in 1a packed inside. The two 6 V lantern batteries can be seen in pockets on each side of the backpack. The total weight of the fully loaded backpack was 24 lbs (approximately 10 kg). Additional free space remained in the backpack.

An analysis is presented that compares quality and yields of DNA extracted from fresh tissue to quality and yields of DNA extracted from tissue exposed to air at 21°C for 48-hour or 72-hours (to simulate transport from the field to the lab). This project was initiated to develop a method that could assure sequence accuracy and could be utilized when other methods produced poor results. First, we tested the field equipment in the lab, and then tested the method with the field equipment under simulated field conditions. All of the equipment can be carried in a standard backpack (Figure 1), in an automobile, on a motorcycle (or bicycle), or by pack animal. Heating of the solutions is accomplished by placing the containers into a pan with water that is warmed with a small gas camp stove, a campfire, or charcoal. Alternatively, the solutions in their containers can be heated directly using a battery-operated heater (several are available commercially). Centrifugation can be performed either with a manually-operated or battery-operated centrifuge. The battery can be in an automobile, motorcycle, lantern, or carried separately. Once the nucleic acids have been extracted and precipitated as CTAB salts or in ethanol as sodium salts, they can be safely transported back to the lab regardless of outside temperature or conditions, without risking additional degradation. Later, the remainder of the extraction process can be completed in a lab.

## Findings

### Centrifuge comparisons

**Figure 2:**
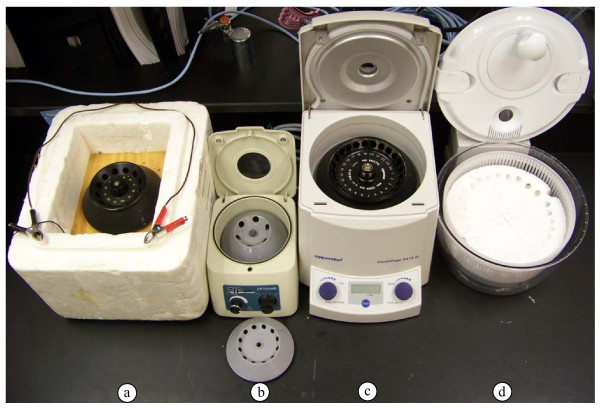
**Microcentrifuges tested. a.** Battery-operated microcentrifuge constructed using a salvaged refrigerator fan motor and a manufactured lightweight centrifuge rotor, mounted in a styrefoam box. **b.** Battery-operated microcentrifuge purchased from a commercial supplier. **c.** Electric (110 V) microcentrifuge. **d.** Manual microcentrifuge constructed from a vegetable spinner with a styrofoam insert to accommodate microcentrifuge tubes (see Methods section for details).

**Figure 3:**
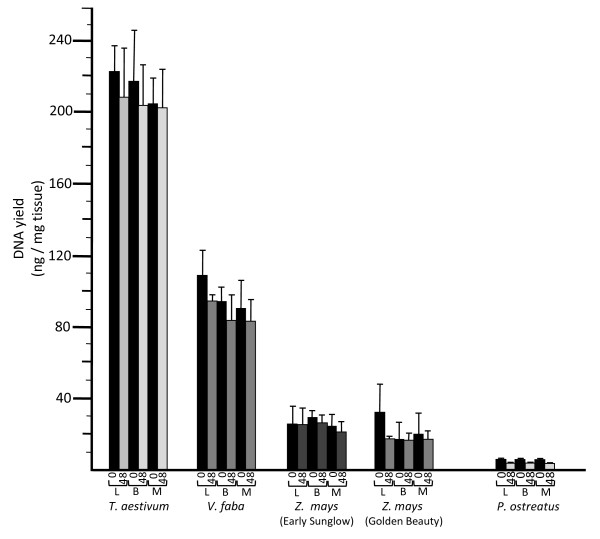
**DNA yields (ng per mg of starting tissue) and DNA condition.** Yields are given for fresh (time = 0 hours) tissues and tissues that had been left at 21°C for 48 hours (time = 48 hours), for each of the three microcentrifuges: electric lab microcentrifuge (L), battery-operated microcentrifuge (B) and manually-operated microcentrifuge (M). DNA condition (degree of degradation) is indicated by shading of the rectangles (bars above indicate one standard deviation, based on sample calculation of means and SD, n = 5). Black rectangles indicate primarily high-molecular weight DNA, medium grays indicate moderate degradation (some diffusion observed for the high-molecular weight band), and light gray indicates extensive degradation (absence of distinct high-molecular weight band, with bright trailing lower molecular weight fluorescence).

DNA was obtained from all species attempted regardless of the type of microcentrifuge that was used (Figures 2 and 3; Table 1). [Note: The one exception to this was for whole seeds of *Cucurbita maxima*. This had been noted in our previous study [3] when the cotyledons were included. However, acceptable yields could be obtained when only the embryonic axes were used.] Yields ranged from approximately 0.3 ng total DNA per mg of starting tissue (ng/mg) to over 200 ng/mg (dependent on tissue type and species). The yields are similar to those from our previous reports [3-5,8,12], which coincidentally ranged from 0.3 to 200 ng/mg. No significant differences in DNA yields resulted when the manual, battery-operated or electric centrifuges were compared (Figure 3).

**Table 1:** DNA extraction results

**Tissues**	**Yield (ng/mg)**		
Species	Fresh^a,b^	48 hour^a,b,c^	72 hour^a,b,d^
**Fungus basidiocarps**			
* Agaricus bispora*	2.0 (−)	4.5 (+/−)	nd
* Pleurotus ostreatus*	1.0 (−)	0.5 (+)	nd
**Plant embryonic axes**			
* Cucurbita maxima*	Y (−)	Y (+/−)	nd
* Picea pungens*	Y (−)	Y (+/−)	nd
* Pinus sylvestris*	Y (−)	Y (+/−)	nd
* Pisum sativum*	Y (−)	Y (+/−)	nd
* Triticum aestivum*	218 (−)	203 (+/−)	nd
* Vicia faba*	92 (−)	82 (+)	nd
* Zea mays*	25 (−)	23 (+)	nd
**Plant embryonic axes with cotyledons**						
* Citrullus lantus*	Y (−)	Y (+/−)	nd			
* Cucumis melo*	Y (−)	Y (+/−)	nd			
* Cucurbita maxima*	0	0	nd			
* Cucurbita pepo*	Y (−)	Y (+/−)	nd			
**Plant leaves**						
* Adiantum capillis-veneris*	2.8 (−)	nd	0			
* Araucaria heterophylla*	6.6 (−)	nd	2.0 (+/−)			
* Chamaedora elegans*	11.1 (−)	nd	2.8 (+/−)			
* Cordyline fructicosa*	3.1 (−)	nd	2.8 (+)			
* Cyperus papyrus*	0.9 (−)	nd	3.9 (+)			
* Davallia fejeensis*	2.2 (−)	nd	7.5 (+/−)			
* Fittonia verschaffeltii*	8.8 (−)	nd	6.8 (+/−)			
* Guzmania lingulata*	5.2 (−)	nd	0			
* Jasmium sambac*	3.6 (−)	nd	2.3 (+)			
* Nemathanthus gregarius*	0.5 (−)	nd	0.3 (+)			
* Picea pungens*	Y (−)	Y (+/−)	nd			
* Pilea nummularifolia*	0.4 (−)	nd	0			
* Pinus sylvestris*	Y (−)	Y (+/−)	nd			
* Populus canadensis*	Y (−)	Y (+/−)	nd			
* Saintpaulia ionantha*	0.3 (−)	nd	0.5 (+)			
**Plant seeds**						
* Capsicum annuum*	Y (−)	Y (+/−)	nd			
* Daucus carrota*	Y (−)	Y (+/−)	nd			
* Solanum lycopersicum*	Y (−)	Y (+/−)	nd			

^a^Samples were dissected, followed by immediate DNA extraction or freezing at −20°C (see text) and then subjected to DNA extraction at a later time. The tissue samples were from 25 mg to 750 mg. DNA amounts were estimated from ethidium bromide fluorescence compared to standards on the same gel. Yields were calculated based on aliquots from the total extracted DNA. The letter “Y” indicates successful extraction where yields were not quantified.

^b^Degree of degradation was based on the sharpness of the high molecular weight band and diffuse fluorescence at lower molecular weights. A plus (+) indicates prominent degradation, a plus/minus (+/−) indicates some degradation, and a minus (−) indicates little or no degradation was evident.

^c^Samples were left out on a laboratory bench top for 48 hours, followed by DNA extraction (plant leaves), or they were frozen at −20°C for DNA extraction at a later time. Not determined = nd.

^d^Samples were left out on a laboratory bench top for 72 hours, followed by DNA extraction. Not determined = nd.

### Fresh versus delayed extraction

**Figure 4:**
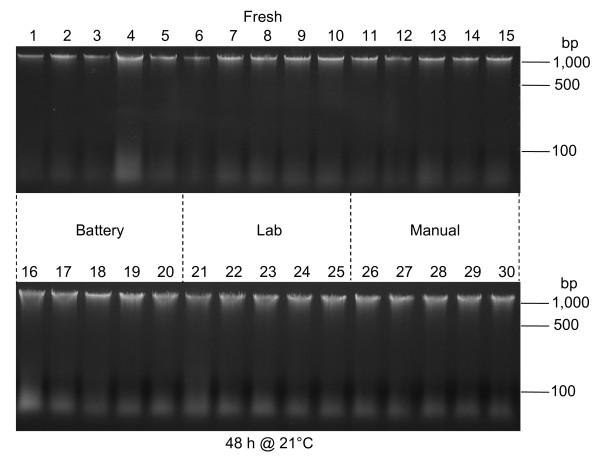
**Examples of DNA degradation in *V. faba *tissues.** Lanes 1–15: DNA extracted individually from 15 embryos dissected from 24-hour imbibed seeds. DNA in lanes 1–5, 6–10, and 11–15 were extracted using the battery-operated, laboratory, and manually-operated microcentrifuges, respectively. Lanes 16–30: DNA extracted individually from 15 embryos dissected from seeds that had been left at 21°C for 48 hours, following 24 hours of imbibition. DNA in lanes 16–20, 21–25, and 26–30 were extracted using the battery-operated, laboratory, and manually-operated microcentrifuges, respectively.

**Figure 5:**
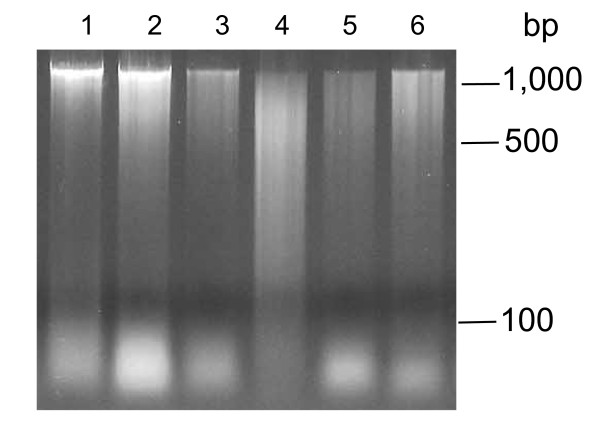
**Example of DNA degradation in *T. aestivum *caryopses.** Lanes 1–3: DNA from fresh caryopses (imbibed for 24 hours). Lanes 4–6: DNA from caryopses left at 21°C for 48 hours (after 24 hours of imbibition). Two of the samples (1 and 4) were extracted using the laboratory microcentrifuge, while four of the samples (2, 3, 5 and 6) were extracted using the manually-operated microcentrifuge.

DNA extracted from fresh samples usually exhibited a prominent high molecular weight band (at approximately 50 kb) on 1% agarose gels, with only a light trailing smear indicating lower molecular weight fragments (Table 1; Figures 4 and 5). DNA from samples that were left on a bench top for 48 or 72 h at 21°C almost always exhibited reduced fluorescence in the high molecular weight band and more prominent lower molecular weight smears (Table 1; Figures 4 and 5). This was most evident for *T. aestivum* embryos (Figure 5), but also was observed for DNA extracted from *Z. mays* embryos, *V. faba* embryos, *P. ostreatus* basidiocarps and the other tissues tested (Table 1; Figure 3 and Figure 4). In some cases the high molecular weight band decreased in intensity by at least 50% (Figure 5), and had become dispersed (i.e., smeared on the gel), indicating a broadening of the distribution of DNA fragment lengths. Also, the fluorescence from lower molecular weight fragments increased.

### Storage in ethanol

Based on agarose gel profiles and PCR amplification, no differences could be detected between extractions taken through the entire extraction process at one time and DNA extractions halted at the ethanol step for one week, and then taken through the remainder of the protocol (data not shown).

### Extraction under simulated field conditions

Results from DNA extraction in the field were similar to extractions in the lab (Table 1). Yields ranged from 0.3 to 200 ng/mg. No major logistical problems were encountered. Set up of the equipment required approximately 30 minutes. With the six-place battery-operated rotor, approximately 30 minutes elapsed from the addition of the hot 2X CTAB buffer through the addition of CTAB precipitation buffer (one potential stopping point). Another 30 minutes elapsed through the addition of the 2.5 volumes of ethanol, for precipitation of the sodium salt of DNA (recommended stopping point). Clean up and repacking of the backpack required approximately 30 minutes.

### PCR amplification

All of the PCR reactions resulted in robust bands consistent with amplicons containing a short portion of the SSU rRNA (small subunit ribosomal RNA) gene, the entire ITS1 (internal transcribed spacer 1), a section the 5.8 S rRNA gene, ITS2 and a short portion of the LSU (large subunit) rRNA gene (data not shown). Amplification was similar for DNAs extracted from fresh samples, as well as those from samples that had been air dried at 21°C for 48 or 72 hours, and those from extracted DNAs that had remained in ethanol for one week at 21°C.

## Discussion

Obtaining intact DNA is key to accurate sequence results. Fresh tissues are the best source for high molecular weight DNA. Freezing with dry ice or liquid nitrogen, or cooling on ice helps to maintain the integrity of the tissue and the DNA, but for remote and hot locations often these are impossible to obtain, transport and maintain. For these locations, time becomes the major limiting factor for slowing nucleic acid degradation. In many cases, rapid desiccation with silica gel has made it possible to transport plant samples back to a lab for successful DNA extraction and analysis [15]. However, for some tissues and species, this method has been less than ideal, leading to unacceptably low yields and/or degraded DNA [17,19]. Storage and transport in saturated NaCl-CTAB also has been used, but again, for some tissues low yields and degradation have been reported, although the addition of ascorbate to the solution improved DNA quality [18,19]. Absorbant paper card technology (e.g., Whatman FTA) is simple fast and inexpensive and has been used successfully for a number or PCR-based applications [16]. However, there have been reports that the resulting DNA is in short fragments, and amplification of regions larger than 1,000 to 2,000 bp might be difficult in some cases because of the short DNA lengths. Also, this technology has not been formulated to protect RNA, and it has been confirmed that RNA is severely degraded when this method is used [27].

Drying racks and ovens have been used extensively for some plant and fungus studies, but some degree of DNA degradation also occurs during the drying process, and little intact RNA remains [8,19]. Previously, we reported that partially and extensively degraded templates can be amplified by PCR, but that often nucleotide changes occur, as evidenced by sequence comparisons with DNA from fresh tissues for the same sample [22-24]. The amount of change primarily is related to tissue type and length of time between tissue sampling and DNA extraction. However, when DNA (as well as RNA) is extracted and precipitated as a salt, it is highly resistant to most forms of degradation. RNA is degraded much more rapidly than DNA, and therefore, in almost all cases it must be extracted (or otherwise preserved) at the time of sampling. One of the best ways to ensure that high quality nucleic acids will be obtained is to minimize the time between sampling, extraction and precipitation as a salt. For field collections, this means performing much of the extraction on location immediately after sampling. Once the nucleic acids are protected as salts free of nucleases and free water, they can be safely stored and transported back to a laboratory without regard to the storage temperature. In fact, commercial suppliers store and ship most of their high quality nucleic acids as dried or lyophilized salts.

Nucleic acids usually begin to degrade shortly after the tissue is sampled from any organism, as the cells begin to die and the cell compartments disintegrate, releasing large quantities of nucleases, as well as proteases and other degradative molecules. These processes also occur during the drying process [8], even when the process is relatively rapid. Therefore, if the DNA is to be used for precise molecular analyses (including sequence comparisons), the degradation processes must be stopped and the nucleic acids must be separated from all nuclease activity and other damaging reactants as soon as possible after sampling. Degradation can take many forms, but enzymatic processes (e.g., by endonucleases and exonucleases), hydrolytic attack (e.g., scission of the backbone phosphodiester bonds, depurination, depyrimidization and deamination), oxidation (e.g., cleavage of the rings of the sugars, guanine, cytosine and thymine), and S-adenosyl methionine transfer of a methyl (e.g., methylation of both purines and pyrimidines) all are common [20,21]. Upon cell damage or death, many of these processes increase in frequency. Additionally, in many regions of the world temperatures often are above 30°C, with high humidity, which increase the rates of degradative reactions for unprotected hydrated nucleic acids.

Most DNA extraction processes break the cells, inactivate nucleases, separate many of the proteins from the nucleic acids, and then precipitate the nucleic acids as salts (e.g., sodium salt of DNA) using ethanol because of their low solubilities in alcohols [1-8]. As salts, nucleic acids are stable at least for years or decades (or longer), and both DNA and RNA often are sold commercially as powdered salts that can be safely shipped and stored for long periods of time at ambient temperatures. Therefore, one of the safest ways to store and transport nucleic acids is to precipitate them as salts in alcohol. They can be safely transported back to the lab either as precipitated salts in alcohol, or as dried salts. Once back in the lab, they can be rehydrated and used immediately for molecular studies, having sustained little or no additional degradation.

For this study, 48- or 72-hour periods of transport back to the lab were simulated prior to extraction. These DNAs were compared to those extracted from tissues that had been sampled immediately prior to the nucleic acid extraction procedure. In all cases, degradation of DNA was minimal or undetected for fresh materials, while some degree of degradation was observed for all tissues that were left at room temperature (21°C) for 48 or 72 hours prior to extraction (Table 1; Figures 3, 4, 5). While yields of DNA (measured as ng of DNA per mg of tissue) were often negatively affected by storage of the tissue for 48 or 72 hours at room temperature, degradation always was greater in DNA extracted from tissues that had remained at room temperature for 48 or 72 hours (Table 1). However, the sodium salt of DNA that remained at the ethanol precipitation step for one week at 21°C exhibited no apparent degradation. Dried salts of DNA are resistant to most degradative processes [25]. DNA from ancient plant, animal, fungal and bacterial samples has been recovered from tissues that have been dehydrated for up to 140 million years, although all exhibit varying degrees of degradation [25]. Therefore, maintenance of a dehydrated state is of prime importance to the preservation of DNA. Dehydrated salts of nucleic acids are among the most degradation resistant forms of these molecules.

RNA is more labile than DNA and RNases can be major problems during attempts to extract intact RNA. However, RNA data can provide an assessment of gene expression levels for developmental, physiological, environmental or other research. Extraction of RNA *in situ* could be invaluable especially for plant and fungus species that cannot be cultivated for study in the lab. We have successfully used the same CTAB extraction method to extract RNA in the lab, although it is best to add an RNase inhibitor to the solutions. Alternatively (and preferably), the TRIzol reagent (which is a mixture of guanidinium thiocyanate, phenol and chloroform; Life Technologies, Carlsbad, CA) method is easily performed in the lab, and should be readily adaptable for use in the field. TRIzol rapidly inactivates nucleases, including RNases [28]. From our experience, good yields of high quality RNA are more often obtained using this method than with the CTAB method. However, the chemicals used are somewhat more hazardous, but can be safely transported and utilized in the field. The TRIzol method can also be used for DNA extraction, but yields are lower than with the CTAB extraction method described. We have successfully used the TRIzol method for RNA extraction and the CTAB extraction method for obtaining DNA and in the lab that we have used for several metagenomic/metabolomic studies of biota (bacteria, viruses, archaea, and eukaryotes, including plants and fungi) in environmental ice samples [29-33]. Therefore, this indicates the possibility of using field extraction methods for metagenomic and other related research projects.

Most items needed to extract nucleic acids in the field are easily transported to, and utilized at, the collection sites. The exceptions are items that require electricity, because sources of electricity are seldom available at the sampling sites. For this study, DNA extraction was performed with three types of microcentrifuges: electric, battery-operated, and manual. Nucleic acids were recovered using all three microcentrifuges for all tissues attempted, and yields were comparable among the three (Figures 3, 4, 5). Therefore, the absence of a power grid or electrical generator is not a barrier to successful nucleic acid extraction. Furthermore, when extractions were performed under simulated field conditions, yields were similar to those obtained from extractions performed in the lab (Table 1). Therefore, extractions performed in the field using the battery-operated microcentrifuge will likely produce results similar to those for extractions of DNA performed in a lab. A source of heat for a water bath is needed during parts of the process. A small propane camp/hiking stove, campfire, charcoal, or other heat source with tripod, pan and water can be used for this. Alternatively, a battery-operated heating unit (used for keeping coffee and tea at 50-60°C) can be used. Some of these attach to laptop computers using a USB port, while others can be operated using other battery sources (including using automobile batteries connected through lighter/accessory sockets). Therefore, alternative sources of power can be readily employed for DNA extractions performed in the field.

**Figure 6:**
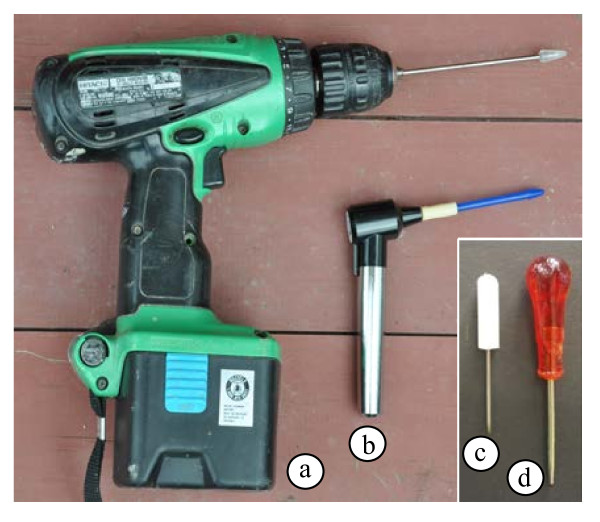
**Grinding tools. a.** Cordless drill (with rechargeable battery) with stainless steel pestle with Teflon end that fits inside a 1.5 ml microcentrifuge tube (purchased commercially). For hard tissues, a stainless steel rod without the Teflon end can be used for grinding. More force can be applied to the sample because of the smaller surface area. **b.** Battery-operated grinder with disposable pestle (purchased commercially). **c.** Manual grinder fashioned from a small screwdriver that has been ground so that the end fits into the bottom of a microfuge tube. **d.** Manual grinder fashioned from an awl that was ground to fit the bottom of microcentrifuge tube. The wider handle distributes the force on ones hand more evenly.

All of the equipment (Figures 1, 2 and 6) needed in the field for nucleic acid extraction can fit within a single average-sized backpack (Figure 1). Because glass bottles are prone to breakage, it is suggested that plastic bottles be used for most reagents. The exceptions are chloroform:isoamyl alcohol and TRIzol. Chloroform dissolves many plastics (especially at high temperatures), but it can be stored in polythene and polypropylene bottles. TRIzol contains phenol and it is best carried in a glass container. Glass bottles should be supported by a box or metal tube to avoid any pressure being applied to the bottle during transport to and from the field. For the field tests, we used a glass bottle placed inside a cardboard screw capped mailing container for the chloroform:isoamyl alcohol (shown in Figure 1). For all procedures, precautions should be taken with each of the hazardous chemicals and procedures. All of the waste should be carried out of the field location for proper disposal. Additionally, local regulations for transport, handling and disposal of any hazardous chemicals must be followed. Static discharges and flames may ignite ethanol, and therefore care should be exercised when working with the ethanol.

Once the tissues of interest are located, the field lab can be set up in about 30 minutes. For DNA extraction, the bottles of 2X CTAB, 5% CTAB (if used) and high-salt TE buffers should be heated first. A temperature of at least 50°C is necessary to assure high yields of DNA, due to lower solubilities of CTAB:DNA salts below 45-50°C. For large, extremely fibrous or hard samples, a cordless drill with a small surface area grinding tip is recommended (Figure 6). However, care must be taken to avoid breaking the bottom of the tube. Small softer samples can be ground in 1.5 ml microcentrifuge tubes directly using a small battery-operated homogenizer or manual grinding tools (Figure 6). Although complete breakage of all portions of the tissue is unnecessary, more cell breakage leads to higher yields. Once the samples have been ground in the hot CTAB, they should be taken through to the alcohol precipitation steps (2.5 volumes of ethanol). However, if time, weather conditions or darkness become issues, it is possible to stop after the first precipitation step (addition of the CTAB precipitation buffer). At this point the nucleic acids are safely complexed with cetyltrimethylammonium and sodium cations. Once back in the lab, the nucleic acids can be carried through the remainder of the extraction and rehydration processes, and assayed by gel electrophoresis for quality and yield. The protocols described should be first attempted in a laboratory using similar tissues to assure that all the procedures can be efficiently, effectively, and safely performed in the field. This method is expected to yield high quality DNA (as well as RNA in some cases) from plant and fungus tissues collected *in situ*.

## Conclusion

The methods and equipment tested yield high quality DNA under field conditions. The field nucleic acid extraction method should prove useful for working in remote sites, where ice, dry ice, or liquid nitrogen are unavailable; when degradation is likely occur due to the long distances between the sample site and the laboratory; when high molecular weight DNA is needed; and for cases where other collection, preservation and extraction methods have been ineffective.

## Methods

### Sample tissues

The following tissues were used in the lab to test the method and field equipment: embyonic axes of *Triticum aestivum (*var*. spelta), Zea mays* (cv. Early Sunglow and cv. Golden Beauty), and *Vicia faba* (cv. Broad Windsor) (caryopses or seeds imbibed at 21°C in sterile reverse osmosis (RO) water for 24 h prior to dissection and extraction); and basidiocarps of *Pleurotus ostreatus. Vicia faba* embryonic axes were selected because from our past experience they consistently yield large quantities of high quality DNA. *Zea mays* embryonic axes were chosen because from our past experience degradation proceeds at rates faster than in *V. faba*. *Triticum aestivum* was chosen for a similar reason, but the rates of degradation were the most rapid of the three species. *Pleurotus ostreatus* was chosen as a representative of fungal basidiocarp tissue. The samples were weighed and then divided into two groups. In group one, the samples were placed into 1.5 ml microcentrifuge tubes and immediately frozen at −20°C. In group two, the tissues were left on a bench top for 48 or 72 hours at 21°C to simulate collection and transport from the field to the lab, and then were placed into 1.5 ml microcentrifuge tubes and frozen at −20°C. A minimum of five replicates was performed for each group of samples.

A second set of tissues was used in a comparison between the electric microcentrifuge and the manually-operated microcentrifuge. These were whole seeds of *Capsicum annuum* (cv California Wonder), *Daucus carrota* (cv Kuroda) and *Solanum lycopersicum (*cvs Beefsteak, Rio Grande, and Rutgers); embryonic axes with coyledons (no seed coats) of *Citrullus lantus* (cv Sugar Baby), *Cucumis melo* (cv Hearts of Gold), *Cucurbita maxima* (cv Black Beauty) and *Cucurbita pepo* (cvs Big Max and Jack O’Lantern); embryonic axes of *Cucurbita maxima* (cv Black Beauty), *Pisum sativum*, *Picea pungens* and *Pinus sylvestris*; leaves of *Populus canadensis*, *Picea pungens* and *Pinus sylvestris*; and basidiocarps of *Agaricus bisporus*. Because of the smaller numbers of extractions per tissue type, quantitation was not performed. All seeds were imbibed for 24 hours (as described above) prior to extraction.

A third set of samples was subjected to extraction under simulated field conditions. Fresh leaf tissues were excised from greenhouse-grown plants from the following species: *Adiantum capillis-veneris*, *Araucaria heterophylla*, *Chamaedora elegans*, *Cordyline frusticosa*, *Cyperus papyrus*, *Davallia fejeensis*, *Fittonia verschaffeltii*, *Guzmania lingulata*, *Jasmium sambac*, *Nemathanthus gregarious*, *Pilea nummularifolia*, and *Saintpaulia ionantha*. Leaves of these species were chosen as representatives of tissue types likely to be sampled in the field. Some had tough (*A. heterophylla, C. elegans*, C. *frusticosa* and *G. lingulata*) and/or waxy leaves *(N. gregarious*), while others were extremely fibrous (*C. papyrus*). A few were fleshy with high water contents (*N. gregarious* and *S. ionantha)*. Two ferns (*A. capillis-veneris* and *D. fejeensis*) were sampled, because DNA from some ferns has been reported to be difficult to extract from field-collected samples [19]. Duplicate samples were collected for each and placed into 1.5 ml microcentrifuge tubes. Samples were weighed for subsequent calculation of yield. Weights were from 27 to 263 mg. One set of samples was subjected immediately to DNA extraction under simulated field conditions (outside, partly sunny, 74°F, light winds). The manual grinding tool (Figure 6d) was used for cell breakage in the hot (55-65°C) 2X CTAB buffer. Between samples, the grinding tool was rinsed with RO water, wiped with a clean tissue and then rinsed with ethanol and wiped with a second clean tissue. Extraction proceeded through the initial step of ethanol precipitation (i.e., addition of 2.5 volumes of 95% ethanol). The other set of samples was left on a lab bench top for 72 hours prior to extraction. They were also extracted under simulated field conditions (outside, mostly sunny, 80°F, light winds) using the same method and equipment.

### DNA extraction

The method is based on previous reports [3-5,8,12]. Briefly, one volume (based on the approximate volume of the tissue) of a hot (55°C to 100°C) 2X buffered CTAB solution [2% CTAB (w/v), 100 mM Tris (pH 8.0), 20 mM EDTA (pH 8.0), 1.4 M NaCl] was added to the tissue. The tissue was ground in the buffer using one of the homogenizers that fits the bottom of the tube (Figure 6). After grinding the tissue in the hot buffer, an equal volume of chloroform:isoamyl alcohol (24:1) was added and the two phases were agitated to form an emulsion. A brief (1–5 min) centrifugation in one of the centrifuges (Figure 3) was used to separate the phases. The upper aqueous phase containing the nucleic acids was moved into a clean microcentrifuge tube, and the chloroform phase was discarded. One-fifth volume of a 5% CTAB solution [5% CTAB (w/v), 0.7 M NaCl] was added to the aqueous phase and thoroughly mixed. [Note: The addition of the 5% CTAB solution can be eliminated in most cases.] A second chloroform:isoamyl extraction was performed. The aqueous phase was moved into a new microfuge tube and one volume of CTAB precipitation buffer [1% CTAB (w/v), 50 mM Tris (pH 8.0), 10 mM EDTA (pH 8.0)] was added, followed by gentle inversion of the tube. This reduces the concentration of sodium cations, which leads to the formation of a CTAB:DNA salt that precipitates at this point. [Note: In the field it is possible to stop at this point and transport the samples back to the lab. However, the preferred stopping point is indicated below.] The tubes were centrifuged for 1–5 min, and the liquid was decanted. High-salt TE buffer [10 mM Tris (pH 8.0), 1 mM EDTA (pH 8.0), 1 M NaCl] was added to each tube to rehydrate the CTAB:DNA pellet (for pellets less than 1 mm in diameter, 100 ul was added; for pellets that were 1–4 mm in diameter, 100–250 ul was added; for larger pellets, up to 500 ul was added). These were heated to facilitate rehydration of the DNA. The high Na^+^ concentration causes a replacement of the cetyltrimethylammonium cations with sodium cations. Sodium salts of nucleic acids have higher solubilities than do cetyltrimethylammonium salts of nucleic acids, thus allowing dissolution of the DNA in the buffer solution. When the pellets were completely dissolved, 2.5 volumes of ethanol (95 or 100%) were added [Note: Similar results have been obtained by adding 0.6 volumes of isopropanol instead of the ethanol]. The tubes were gently inverted several times to mix the two liquids and precipitate the sodium salt of DNA. [Note: In the field, this is the preferred stopping point. The sodium salt of DNA in ethanol can be safely transported for further processing in a laboratory. DNA can be stored for years this way without detectable degradation. Parafilm can be placed around the top of the tube to protect from spillage, or screw-capped tubes can be used. The remainder of the procedure can be performed in a laboratory where a freezer is available for storage of the hydrated DNA.] In the lab, the nucleic acids were pelleted by centrifugation for 1–5 min. The liquid was carefully decanted and the pellets were washed once with 200 ul of 80% ethanol. This was followed by a 1 min centrifugation to assure that the pellets were at the bottom of the tube. The ethanol was decanted and the pellets were allowed to air dry or were dried in a vacuum centrifuge. [Note: The pellet should not be completely dried, because this can lead to difficult rehydration.] The nucleic acids were rehydrated in 0.1X TE [1 mM Tris (pH 8.0), 0.1 mM EDTA (pH 8.0); for pellets smaller than 1 mm in diameter, 20 ul was added; up to 100 ul was added to larger pellets].

### Gel electrophoresis and yield calculations

The DNA was subjected to electrophoresis at 5 V/cm for 1–2 h on 1% agarose gels in TBE (89 mM Tris-base, 89 mM borate, 2 mM EDTA, pH 8.0), containing 0.5 ug/ml ethidium bromide. The fluorescence from UV illumination of each gel was digitally photographed. DNAs were compared to standards loaded on the same gel in order to assess quality and quantity of DNA. Quality was indicated by the amount of fluorescence in the high molecular weight band, compared to fluorescence in lower molecular weight regions of the gels. Quantity was estimated by comparing the molecular weight standards (specific amounts loaded onto the gels) with the total amount of fluorescence in each sample lane. These amounts were used to calculate the total amount of DNA extracted from each sample. Yields were calculated for each sample by dividing the total amount of DNA (in ng) by the amount of starting tissue (in mg).

### PCR amplification

Many of the extracted DNAs were tested using PCR amplification. Briefly, approximately 1–10 ng of each DNA was used in a reaction mix, using a GeneAmp PCR Reagent Kit (Applied Biosystems, Branchburg, New Jersey). Each reaction consisted of 50 pmol of each primer (ITS4 and ITS5, [34]), 10 pmol of each dNTP, 2U *Taq* DNA polymerase, 50 mM KCl, 1.5 mM MgCl_2_, in a total volume of 25 μl. The thermal cycler (Mastercycler gradient, Eppendorf, Westbury, NY) program used was: 94°C for 2 min; 40 cycles of 1 min at 94°C, 2 min at 55°C, a ramp of 1°C per 8 sec, and then 2 min at 72°C; followed by an incubation for 10 min at 72°C. PCR reactions were subjected to electrophoresis on 1.0% agarose gels (as above) and photographed (as above).

### Storage of DNA in ethanol

Twelve randomly selected tissue samples were divided into two tubes. Extraction was completed for one set through the addition of 0.1X TE. For the other set, extraction was stopped after addition of the 2.5 volumes of ethanol. These were allowed to remain at 21°C for one week. Subsequently, extraction was completed. The DNAs were compared on 1% agarose gels (as above) and by PCR (as above).

### Centrifuges

Four different centrifuges were used for the initial comparisons (Figure 2). One of these was manufactured in our lab, using a 12 V motor from a salvaged refrigerator fan. The shaft was altered to accept a lightweight microfuge rotor that was purchased commercially, and the entire apparatus was mounted onto a piece of plywood cut to fit into a styrofoam box (Figure 2a). The centrifuge was capable of approximately 8,000 rpm (rcf = 5,000×g) when fully loaded with rotor and microcentrifuge tubes, and supplied with 12 V of power from two 6 V lantern batteries (wired in series). The second, a Zip Spin ZS-1 (Figure 2b), was purchased commercially (LW Scientific, Lawrenceville, GA), is capable of speeds up to 7,000 rpm (rcf = 3,000×g). Power was supplied with two 6 V lantern batteries (wired in series), AC adapter, automobile battery or automobile lighter/accessory adapter. The second centrifuge (with lantern batteries as the power source) was used for all of the extractions performed under simulated field conditions. The third device was a 110 V microcentrifuge (model 5415D; Eppendorf, Hauppauge, NY; Figure 2c), capable of speeds up to 13,200 rpm (rcf = 16,000×g), which was used as an example of a standard laboratory microcentrifuge. The final centrifuge was manually operated. It was constructed in our lab starting with a “Salad Spinner” (Model X70002, Xtraordinary Home Products LLC, Lincolnshire, IL), a kitchen device designed to remove water from vegetables by centrifugal force. A circular styrofoam insert that snugly fit inside the inner basket was manufactured in our lab (Figure 2d). Holes were cut at a 45° angle into the styrofoam with cork borers to accommodate microcentrifuge tubes. While there are several manufacturers of manual salad centrifuges, this one was geared such that with each complete turn of the handle, the inner basket made five complete revolutions. A high gearing ratio is important, because a speed of approximately 1,000 rpm (rcf = 120×g) can be attained with this device.

## Abbreviations

bp: Base pairs; CTAB: Cetyltrimethylammonium bromide (synonym, hexadecyltrimethylammonium bromide); dNTP: Deoxynucleoside triphosphate; EDTA: Ethylenediaminetetraacetic acid; ITS1: ITS2, internal transcribed spacer 1, internal transcribed spacer 2; kb: Kilobases; LSU: Large subunit; mg: Milligrams; NaCl: Sodium chloride; ng: Nanograms; PCR: Polymerase chain reaction; RO: Reverse osmosis; rcf: Relative centrifugal force; rRNA: Ribosomal RNA; SSU: Small subunit; Tris: Tris(hydroxymethyl)aminomethane; USB: Universal serial bus.

## Competing interests

The authors declare that they have no competing interests.

## Authors’ contributions

SR designed the study, the DNA extraction procedure and backpack field DNA extraction kit, and instructed/supervised all other authors. AA, YS and SR designed and constructed some of the equipment. AA, KH and HC performed most of the DNA extractions. SR performed all of the DNA extractions under simulated field conditions. AA performed all of the PCR amplification procedures, as well as the experiments on storage of DNA in ethanol. AA and SR performed most of the data analyses, and wrote the initial drafts of the manuscript. All authors have read and approved the final manuscript.
